# Response Surface Methodology for the Optimization of Celecoxib Self-microemulsifying Drug delivery System

**DOI:** 10.4103/0250-474X.45395

**Published:** 2008

**Authors:** Jessy Shaji, Shital Lodha

**Affiliations:** Pharmaceutics Department, Principal K. M. Kundanani College of Pharmacy, 23, Jote Joy Bldg, Rambhau Salgaokar Marg, Colaba, Mumbai-400 005, India

**Keywords:** Self microemulsifying drug delivery system, particle size, celecoxib, optimization, response surface methodology

## Abstract

The aim of the present study was to prepare, evaluate and optimize, self micro emulsifying drug delivery system of celecoxib. A 3 factor, 3 level factorial design was used for the optimization procedure with different amounts of Labrafil 2609 WL, Labrasol, and Cremophor EL as the independent variables. The response variable was selected on particle size (nm) of the droplets after dilution in 0.1N HCl. Particle size of the self micro-emulsifying drug delivery system depends on the quantity of above three independent variables. Three different levels of each independent variable were selected for the optimization. Mathematical equation and response surface plots were used to relate the dependent and independent variables. The regression equation generated for the particle size after dilution was, Particle size (Y)= +27.83+76.07×A-23.62×B-43.83×C+52.72×A^2^+9.82×B^2^+27.20×C^2^-14.52×A×B-32.38×A×C+12.1×B×C, where, A=Labrafil 2609 WL, B= Labrasol, C= Cremophor EL, Y= particle size. The optimized model predicted a particle size of 28.33 nm with 0.16ml of labrafil 2609 WL, 0.17ml Labrasol and 0.22ml of Cremophor EL. The observed response were in close agreement with the predicted values of the optimized formulation. This demonstrates the reliability of the optimization procedure in predicting particle size of self microemulsifying delivery system for celecoxib.

Approximately 35-40% of new drug candidates have poor water solubility. The oral delivery of such drugs is frequently associated with low bioavailability, high inter-subject and intra-subject variability and lack of dose proportionality. Efforts are needed to enhance the oral bioavailability of class-2 and class-4 lipophillic drugs in order to increase the clinical efficacy. Currently various formulation strategies are in practice such as use of cyclodextrin, nanoparticles, solid dispersion, micronization, liposomes, permeation enhancers, lipid solutions and microemulsions^1^,^2^.

Microemulsion has got advantages like excellent thermodynamic stability, high drug solubilization capacity, improved oral bioavailability and protection against enzymatic hydrolysis. The only problem with microemulsion is poor palatability due to the lipid content leading to the poor patient compliance. Moreover due to their water content, microemulsion cannot be encapsulated in soft gelatin and hard gelatin capsules; hence there is a need for anhydrous self-micro emulsifying drug delivery system^3^.

Compared to microemulsion, Self-microemolsilying Drug delivery System (SMEDDS) offers advantages such as, improved physical stability profile upon long term storage, volume considerations and can be presented in concentrated form and can be filled directly into soft or hard gelatin capsules for convenient oral delivery. The most significant one is that microemulsion dosage form uses a large amount of surfactants for the purpose of forming microemulsions. This has posed clinical liabilities as surfactants often have potential toxic effects when used at high levels. The use of cosolvent/cosurfactants is believed to act as a good solubilizer for both water and oil and reduce surface tension by stabilizing film formation between the two phases at sufficiently low concentration. This rules out the use of the large amount of surfactants in the formation of microemulsion.

Self-micro emulsifying system is isotropic mixture of oil, surfactant and hydrophilic co-surfactant which forms fine o/w emulsion, when introduced in excess of aqueous phase under conditions of gentle agitation^3^. Agitation will be provided by body movement and gastrointestinal movement *in vivo*. Bases for self-micro emulsifying system have been formulated using medium chain tri-glyceride oils and non-ionic surfactant, which are acceptable for oral ingestion^4^.

The lipophillic (poorly water soluble) drugs such as celecoxib and other drugs of same property are nifedipine, griseofulvin, cyclosporine, digoxin, itraconazole, carbamazepine, piroxicam, fluconazole, indomethacin, steroids, ibuprofen, diazepam, finasteroids, difunisal etc., are formulated in self micro-emulsifying drug delivery system (SMEDDS) to improve efficacy and safety.

A literature search reveals an exhaustive number of publications characterizing the self emulsified drug delivery system^5^–^12^. Reported studies use different methods for *In vitro* evaluation such as self emulsification time, cumulative percent release, low frequency dielectric spectroscopy, zeta potential measurement and surface tensiometry. Particle size of SMEDDS after dilution was selected as criteria for *In vitro* evaluation. Smaller the particle size of SMEDDS more is the release of drug with better bioavailability. Particle size of around 20 nm gives total transparent system upon dilution, which acts as a solution. So, particle size was selected as criteria for the optimization. Screening and optimizing self-emulsified drug delivery system could be further simplified by the use of statistical design that requires only a small number of experiments, thereby eliminating the need for time-consuming and detailed ternary phase diagrams. The statistical optimization design has been documented for the formulation of pharmaceutical solid dosage forms^13^–^15^. Wehrle *et al.*^16^ have reported the use of a sequential statistical design for optimizing the droplet size of a miconazole emulsion. Here SMEDDS were tried to optimize on the basis of particle size after dilution in 0.1N HCl, which are profoundly influenced by several formulation variables.

Celecoxib is a nonsteroidal antiinflammatory drug which selectively inhibits the enzyme, cyclo-oxygenase-2 (COX-2). Celecoxib occurs as an odorless, white to off-white crystalline powder. The aqueous solubility of celecoxib at a pH less than 9 is about 5 μg/ml at 5–40°. Doses given for celecoxib in different indications range from 50-400 mg OD/BD. It is difficult for a drug to get dissolved in a limited volume of the acidic content in the stomach; hence celecoxib shows dissolution limited bioavailability^17^–^19^. It is a class-2 drug with poor solubility and high permeability through out the GIT. The bioavailability of celecoxib was found to be 22-40% when given in a capsule form. So celecoxib was taken as a model drug for the improvement of bioavailability^20^.

The objective of the present work was to apply response surface methodology for the optimization of celecoxib self-micro emulsifying drug delivery system. As a part of optimization process, the main effects, interaction effects and the quadratic effects of the formulation ingredients were investigated. Excipients and their interaction were evaluated for their effect on the particle size of celecoxib SMEDDS after dilution. Particle size is particularly important since release rates are greatly influenced by particle size. Particle size is affected by formulation ingredients.

## MATERIALS AND METHODS

Celecoxib was a generous gift from Alkem Pvt Ltd. (Mumbai, India) Labrafil 2609 WL, labrasol, transcutol were generous gift from Colorcon Asia Pvt Ltd. (Goa, India). Cremophor EL was generous gift from BASF (Mumbai, India). Transparent hard gelatin capsules were gifted from Associated Capsule Group (Dhahanu, India).

### Experimental design:

Optimization of the celecoxib SMEDDS were done using 3 level factorial design. From the preliminary study Labrafil WL 2609 was selected as lipophile, Labrasol as a surfactant and Cremophor EL as a co-surfactant. Quantities of Labrafil 2609, Labrasol and Cremophor EL were selected as the three factors for optimization. Three levels for each factor were used to construct experimental design. Levels for Labrafil WL 2609 (0.1, 0.2, 0.3), Labrasol (0.1, 0.2, 0.3) and Cremophor EL (0.15, 0.25, 0.35) were selected from preliminary study. Particle size was selected as a desire to response for optimization. 32 experiments were planned as per 3^3^ factorial designs.

Since there are only 3 levels for each factor, the appropriate model is the quadratic model. The non-linear quadratic model generated by the design was: Y= +27.83+76.07×A-23.62×B-43.83×C+52.72×A^2^+9.82×B^2^+27.20×C^2^-14.52×A×B-32.38×A×C+12.1×B×C. Response surface diagram was constructed using Design Expert 6 version. Formulation was optimized with the help of response surface diagram.

### Preparation of the celecoxib self micro emulsifying formulation:

Accurately weighed 50 mg of celecoxib was mixed with 50 μl of transcutol in a borosil glass test tube. Labrafil 2609 WL, labrasol and cremophor EL were added with the help of a positive displacement pipette and mixed on a cyclomixer to get a uniform mixture. Prepared formulations were filled in a transparent gelatin capsule (Size “0”). Capsule was sealed with the help of gelatin band to avoid leakage. Each capsule represented 50 mg of Celecoxib with 50 μl of transcutol in addition to the specified amount of labrafil 2609 WL, labrasol and cremophor EL as given in Table 1.

**TABLE 1:** VARIABLES AND LEVELS FOR 3 LEVELS, 3 FACTORIAL DESIGN.

Independent Variables	Levels		
	
	Low	Middle	High
A- Amount of Labrafil 2609 WL in ml	0.1	0.2	0.3
B- Amount of Labrasol in ml	0.1	0.2	0.3
C- Amount of Cremophor EL in ml	0.15	0.25	0.35
**Dependent variable:** Y= Particle size of the droplets after dilution in the 0.1N HCL			

The independent factors and the dependent variables used in the design are listed, where Y=particle size, A=Quantity in ml for Labrafil 2609 WL, B= Quantity in ml for Labrasol and C= Quantity in ml for Cremophor EL.

Particle size of the self-microemulsified formulation filled in the capsule was determined using Coulter N4 Plus at 20°. The capsule was placed in 200 ml of 0.1N HCl. It was allowed to disintegrate for 5 min. Particle size was measured using the following parameters. Profile: Unimodel <2% CV<1000 nm, angle selected: 90.0, run time: 425 s, equilibrations time: 2 min, repetition: repeat 1 time for each angle.

## RESULTS AND DISCUSSION

Microemulsion region was found out by constructing pseudoternary phase diagrams. Approximate quantities of lipophiles (30%) and surfactant system (above 50%) were decided. Further optimization of the formulation was done by Design Expert software. 32 experiments were planned as per 3^3^ factorial designs. Particle size of SMEDDS was selected as a response for optimization. The experimental runs and the observed response for the 32 formulations are given in Table 2. Based on the experimental design, the factor combination resulted in different particle size. The range of response was 334.3 nm in standard run 3 (maximum) and 13.5 nm in standard run 22 (minimum). The mathematics in the form of a polynomial equation for the measured response was obtained with statistical package Design expert® version6 software for the experiment design is listed in Table 3. The polynomial equation relating the response Y and variables A,B,C as: Y=+27.83+76.07×A-23.62×B-43.83×C+52.72×A^2^+9.82×B^2^+27.20×C^2^-14.52×A×B-32.38×A×C+ 12.1×B×C, where, Y= particle size, A= quantity in ml for Labrafil 2609 WL, B= quantity in ml for Labrasol and C= quantity in ml for cremophor EL.

**TABLE 2:** EXPERIMENTAL DESIGN AND RESPONSE FOR 3 LEVEL, 3 FACTORIAL DESIGN PARTICLE SIZE ANALYSIS

Standard Run	Factor-1 A	Factor-2 B	Factor-3 C	Response Particle size (nm)
16	0.1	0.3	0.25	20.2
26	0.2	0.3	0.35	16.2
1	0.1	0.1	0.15	60
11	0.2	0.1	0.25	49.6
17	0.2	0.3	0.25	23.8
22	0.1	0.2	0.35	13.5
23	0.2	0.2	0.35	21.2
28	0.2	0.2	0.25	19.9
12	0.3	0.1	0.25	195.4
6	0.3	0.2	0.15	240
13	0.1	0.2	0.25	32
4	0.1	0.2	0.15	48.4
25	0.1	0.3	0.35	16.9
14	0.2	0.2	0.25	19.6
10	0.1	0.1	0.25	22.4
5	0.2	0.2	0.15	103.6
2	0.2	0.1	0.15	164.4
24	0.3	0.2	0.35	130
32	0.2	0.2	0.25	21
3	0.3	0.1	0.15	334.3
9	0.3	0.3	0.15	228.6
20	0.2	0.1	0.35	47
19	0.1	0.1	0.35	24.7
29	0.2	0.2	0.25	22.2
8	0.2	0.3	0.15	61.3
7	0.1	0.3	0.15	24.4
15	0.3	0.2	0.25	155.8
21	0.3	0.1	0.35	133.2
27	0.3	0.3	0.35	73.4
30	0.2	0.2	0.25	20.5
18	0.3	0.3	0.25	141
31	0.2	0.2	0.25	21.5

A= Labrafil 2609 WL (ml), B= Labrasol (ml), C= Cremophor EL (ml)

**TABLE 3:** OPTIMIZED VALUES OBTAINED BY APPLYING CONSTRAINTS ON VARIABLES AND RESPONSE

Variables	Quantity (ml)	Expected particle size*	Observed Particle size*
A-Labrafil 2609WL	0.16		
B-Labrasol	0.17	24.87	28.33
C-Cremophor EL	0.22		

A-Labrafil 2609WL, B-Labrasol, C-Cremophor EL

* particle size in nm.

**Fig. 1:**
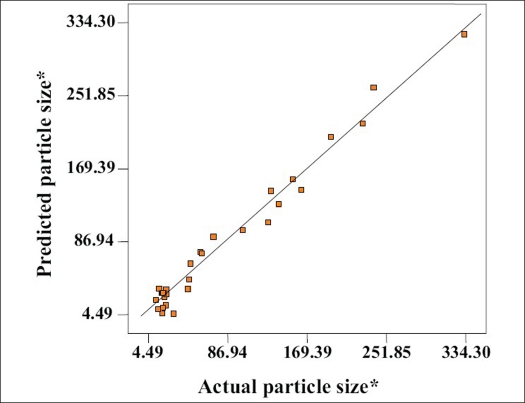
Predicted Vs Actual particle size. *Particle size in nanometers

The above equation represents the quantitative effect of process variables (A, B, C) and their interaction on the response (Y). The values of the coefficient A, B and C are related to the effect of these variables on the response Y. Coefficient with more than one factor term and those with higher order terms represent interaction term. A positive sign represent a synergistic effect, while a negative sign indicate an antagonistic effect. The values of the coefficient A, B and C were substituted in the equation to obtain the theoretical values of Y. The theoretical (predicted) and the observed values were in resonably good agreement as seen from fig. 1.

The significance of the ratio of mean square variation due to regression and residual error was tested using analysis of variance (ANOVA). The ANOVA indicated a significant (P< 0.05) effect of factors on response.

**Fig. 2:**
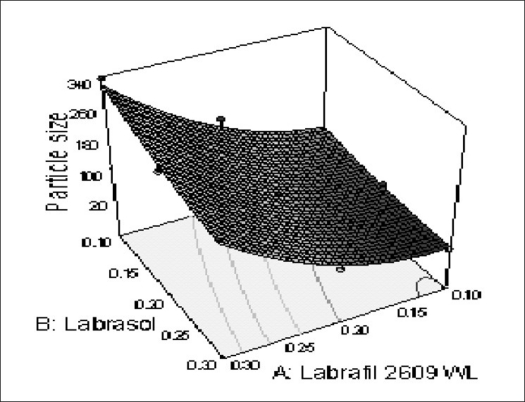
Response surface plot (3D) of the effect of the amount of A and B added on the response Y X1=A: Labrafil 2609 WL, X2=B: Labrasol, Actual factor C: Cremophor EL= 0.15.

**Fig. 3:**
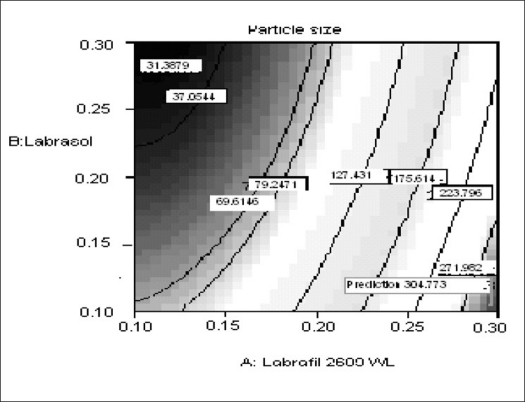
Contour plot of the effect of the amount of A and B on the response of Y X1=A: Labrafil 2609 WL, X2=B: Labrasol, Actual factor C: Cremophor EL= 0.15.

The relationship between the dependent and independent variables was further elucidated using contour and response surface plots. The effects of A (labrafil 2609 WL) and B (labrasol) and their interaction on Y (Particle size) at a fixed level of C (cremophor EL) are given in figs. 2 and 3. At low level of B (Amount of labrasol added) Y increases from 74.72 nm to 314.03 nm when amount A increases from 0.1 ml to 0.3 ml. Similarly, at high level of B, Y increases from 31.6 nm to 219.53 nm when A increases from 0.1 to 0.3 ml as shown in fig. 3.

**Fig. 4:**
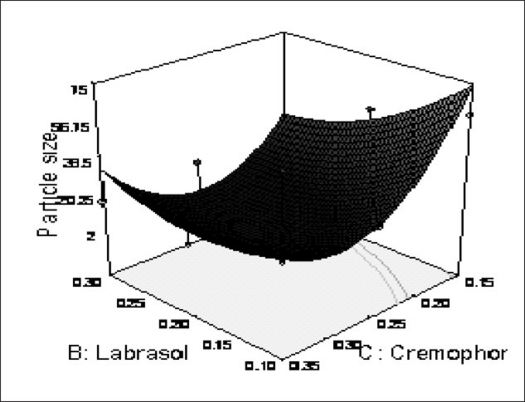
Response surface plot (3D) of the effect of the amount of B and C added on the response Y X1=A: Labrafil 2609 WL, X2=B: Labrasol, Actual factor C: Cremophor EL= 0.15.

**Fig. 5:**
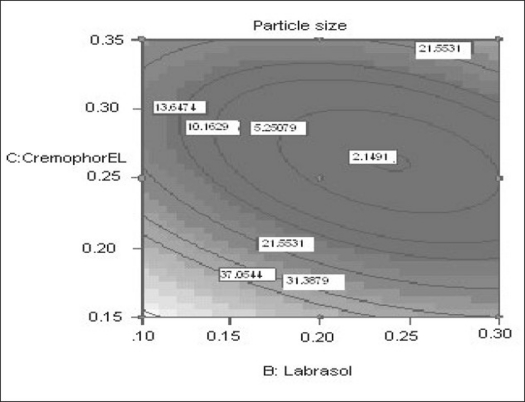
Contour plot showing the effect of the amount of B and C on the response Y X1= B: Labrasol, X2= C: Cremophor EL, Actual Factor= Labrafil 2609 WL= 0.10.

The possible explanation for this is that at higher concentration of lipophile and with a low amount of added labrasol the proportion of surfactant that facilitate water penetration decreases, and the mixture becomes more lipophilic causing increase difficulty in emulsification, hence increase in the particle size^21^. This was shown in the figs. 4 and 5. When effect of B, C and their interaction on Y at a fixed level of A was observed, it was found that, at low level of B (amount of labrasol), Y decreases from 74.4 nm to 26.99 nm, as amount of cremophor EL increases from 0.15 to 0.35 ml. At higher level of B, Y remains approximately constant (32 nm) at level of 0.15 and 0.35 ml of C but decrease to 4.93 nm at 0.25 ml. The possible explanation for this is that cremophor EL (surfactant) strongly localized at the surface of the emulsion droplet reduces interface free energy and provide a mechanical barrier to coalescence resulting in a thermodynamically spontaneous dispersion^22^. However, at high cremophor EL concentration because of its high viscosity, progress of efficient emulsification may be compromised due to viscous liquid crystalline gel forming at the surfactant-water interface.

**Fig. 6:**
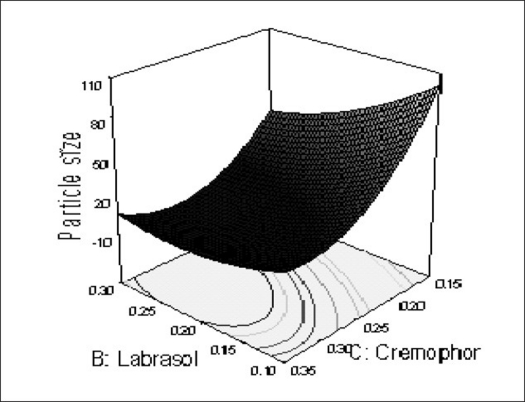
Contour plot showing the effect of the amount of B and C on the response Y X1= B: Labrasol, X2= C: Cremophor EL, Actual Factor= Labrafil 2609 WL= 0.16 at a fixed value of A (0.16 ml).

The effect of B and C and their interaction on Y at a fixed level of A are given in figs. 4 and 5. At low level of C (0.15 ml cremophor EL), Y decrease from 74.21 nm to 31.68 nm as the amount of Labrasol increases from 0.1 to 0.3 ml. At middle level of C, Y changed from 23.40 nm to 4 nm to 5.20 nm, as B changed from 0.1 to 0.2 to 0.3 ml. At high level of C, Y changes from 26.18 nm to 19 nm to 31 nm, as B changed from 0.1 to 0.2 to 0.3 ml. Particle size of the droplet was found minimum (2.11 nm) at a ratio of Cremophor EL to labrasol 1.125, when labrafil 2609 WL was at lower level (Fig. 6). Above results can be explained on the basis of required HLB value. Here quantities of labrasol and Cremophor EL are critical. Microemulsion gives minimum particle size at a critical ratio of surfactant system^23^. Quantities of surfactants at this level provide required HLB value to emulsify lipophile and give microemulsion of lowest particle size.

**Fig. 7:**
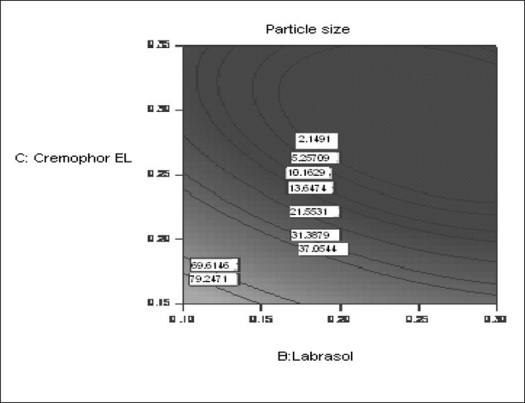
Response surface plot (3D) Showing the effect of the amount of B and C added on the response Y X1= B: Labrasol, X2= C: Cremophor EL, Actual Factor= Labrafil 2609 WL= 0.10 at a fix value of A (0.16 ml).

After generating the polynomial equation relating the dependent and independent variables, the process was optimized for the response Y. Optimization was performed to obtain the levels of A, B and C, which minimize Y. Quantity of A, B and C was selected, which was suitable to be filled in a “0” size hard gelatin capsule. Quantity of A should not be less then 0.160 ml otherwise drug may precipitate out from the formulation. Figs. 6 and 7 show response surface diagram (3D). Particle size of the optimized formulation below 30 nm was taken as a constraint for Y. To verify these values, a new formulation was prepared according to the predicted levels of A, B and C. Obtained Y was in close agreement with the predicted value. The predicted and observed values are shown in Table 3. This demonstrates reliability of the optimization procedure in predicting the particle size of SMEDDS.

Optimization of the self microemulsifying formulation of celecoxib was performed using 3 factor, 3 level design. The amount of added A (labrafil 2609 WL), B (labrasol) and C (cremophor EL) showed significant effect on the particle size and physical appearance of the resultant microemulsion on dilution in 0.1N HCl. The quantitative effect of these factors at different levels was predicted using polynomial equation. Response methodology was then used to predict the levels of the factor A, B and C required to obtain an optimum formulation with particle size bellow 30 nm. A new formulation was prepared according to these levels. Observed response was in close agreement with the predicted values of the optimized formulation, thereby demonstrating the feasibility of the optimization procedure in developing self micro emulsifying delivery of celecoxib.
